# Death and Dying: Boundaries and Roles of Families and Healthcare Workers During Patient End of Life

**DOI:** 10.1093/geroni/igab046.2852

**Published:** 2021-12-17

**Authors:** Amber Thompson, Rebecca Utz

**Affiliations:** University of Utah, Salt Lake City, Utah, United States

## Abstract

While death and dying often occur within or adjacent to the healthcare setting, grief & support of patients at end-of-life (EOL) remain largely within the realm of the family. Given this division of roles, healthcare workers intentionally set professional boundaries that balance their need to be empathetic and compassionate for patient and their families during EOL, while also maintaining a sense of objectivity and detachment which allows them to cope with patient loss and manage the competing demands of their workday. Tensions occur when healthcare workers are required to cross boundaries at EOL, either voluntarily or involuntarily. Using unobtrusive digital ethnography of a publicly accessible online forum for healthcare providers, this research investigates the boundaries set by families and healthcare workers at EOL, and how EOL circumstances sometimes require healthcare workers to cross or violate these professional boundaries. We suggest that the needs of the family at EOL (not necessarily the patient) serve as the catalyst for both boundary crossing & boundary violations for healthcare workers. Our data reveal that (1) boundary setting and training ought to address the patient-physician-family relationship (not just patient-physician), since the family members are such an integral part of EOL; (2) these EOL dynamics apply beyond the physician and should include all healthcare workers (nurses, etc.). As a result, patient & family centered care may not be fully achieved at EOL due to the ambiguity in the expected roles played by both families and healthcare workers during patient death and dying.

